# Effects of increasing omega-3 fatty acids on growth performance, immune response, and mortality in nursery pigs

**DOI:** 10.1093/tas/txae002

**Published:** 2024-01-05

**Authors:** Jenna J Bromm, Mike D Tokach, Jason C Woodworth, Robert D Goodband, Joel M DeRouchey, Chad W Hastad, Zach B Post, Josh R Flohr, Raymond A Schmitt, Jose F Zarate Ledesma, Jordan T Gebhardt

**Affiliations:** Department of Animal Sciences and Industry, College of Agriculture, Kansas State University, Manhattan, KS 66506, USA; Department of Animal Sciences and Industry, College of Agriculture, Kansas State University, Manhattan, KS 66506, USA; Department of Animal Sciences and Industry, College of Agriculture, Kansas State University, Manhattan, KS 66506, USA; Department of Animal Sciences and Industry, College of Agriculture, Kansas State University, Manhattan, KS 66506, USA; Department of Animal Sciences and Industry, College of Agriculture, Kansas State University, Manhattan, KS 66506, USA; New Fashion Pork, Jackson, MN 56143, USA; New Fashion Pork, Jackson, MN 56143, USA; Seaboard Foods, Guymon, OK 73942, USA; Seaboard Foods, Guymon, OK 73942, USA; Seaboard Foods, Guymon, OK 73942, USA; Department of Diagnostic Medicine/Pathobiology, College of Veterinary Medicine, Kansas State University, Manhattan, KS 66506, USA

**Keywords:** immune response, LPS, mortality, nursery pigs, omega-3, PRRSV

## Abstract

Three experiments evaluated omega-3 fatty acids, provided by O3 trial feed, on nursery pig growth performance, mortality, and response to an LPS immune challenge or natural Porcine reproductive and respiratory virus (PRRSV) outbreak. In experiment 1, 350 pigs (241 × 600, DNA; initially 5.8 kg) were used. Pens of pigs were randomly assigned to one of the five dietary treatments containing increasing omega-3 fatty acids (0%, 1%, 2%, 3%, and 4% O3 trial feed) with 14 replications per treatment. On day 25, two pigs per pen were injected intramuscularly with 20 μg *Escherichia coli* LPS per kg BW and one pig per pen was injected with saline as a control. Body temperature was taken from all three pigs prior to and 2, 4, 6, and 12 h post-LPS challenge. Serum IL-1β and TNF-α concentrations were determined in LPS-challenged pigs 24 h prior and 4 h post-LPS challenge. There was no interaction between treatment and time for change in body temperature (*P* > 0.10). Overall, increasing the O3 trial feed did not affect (*P* > 0.10) ADG, ADFI, G:F, IL-1β, or TNF-α. In experiment 2, 1,056 pigs (PIC TR4 × [Fast LW × PIC L02] initially 7.3 kg) were used. Pens of pigs were randomly assigned to one of the four dietary treatments containing increasing omega-3 fatty acids (0%, 0.75%, 1.5%, and 3% O3 trial feed) with 12 replications per treatment. Oral fluids tested negative on days 7 and 14, but then positive for North American PRRSV virus via PCR on days 21, 28, 35, and 42. Overall, increasing O3 trial feed increased (linear, *P* < 0.001) ADG, ADFI, and G:F and decreased (linear, *P* = 0.027) total removals and mortality. In experiment 3, 91,140 pigs (DNA 600 × PIC 1050; initially 5.1 kg), originating from PRRSV-positive sow farms, were used across eight nursery sites. Each site contained five barns with two rooms per barn and ~1,100 pigs per room. Rooms of pigs were blocked by nursery site and allocated within sow source to one of the two dietary treatments (control or 3% O3 trial feed) with 40 replications per treatment. Oral fluids from 61 of the 80 rooms tested positive for North American PRRSV virus 1 wk postweaning and 78 of the 80 rooms tested positive 3 wk after weaning. Overall, O3 trial feed did not affect ADG, ADFI, or G:F but increased (*P* < 0.001) total removals and mortalities. In summary, increasing omega-3 fatty acids, sourced by O3 trial feed, did not improve growth performance or immune response in healthy pigs given an LPS challenge. However, it appears that if omega-3 fatty acids are fed prior to a natural PRRSV break (as in experiment 2), growth performance may be improved, and mortality reduced.

## Teaser

Increasing the level of omega-3 fatty acids in the diet before a natural PRRSV break, through the inclusion of O3 trial feed, can improve growth performance, immune response, and reduce mortality during a health challenge.

## Introduction

The need to better understand the interaction between nutrition and the immune system is critical to reach peak production efficiency under disease situations. During a disease challenge, an animal will redirect nutrients away from growth toward the immune response (Klasing and Leshchinsky, 2000; Caroll et al., 2003). Including omega-3 fatty acids in the diet during a disease challenge may alter allocation of nutrients to improve pig performance (Liu et al., 2003; Duan et al., 2014).

Inclusion of omega-3 fatty acids in the diet has been used as a nutritional strategy to improve the immune response by reducing the omega-6:3 fatty acid ratio. Observations from previous research show that lowering the ratio of omega-6:3 from the 10:1 or 20:1 found in typical swine diets, to a range of 3:1 or 5:1 improves immune response (Duan et al., 2014; Huber et al., 2018). Reducing the omega-6:3 fatty acid ratio increases the incorporation of omega-3 fatty acids into cell membranes to make it available for improved immune function during an immune challenge (Huber et al., 2018). Health challenges within swine production can take a variety of forms, including multifactorial disease processes including bacterial and/or viral pathogens. Currently, there is little information known about the potential immunomodulatory effects of reducing the omega-6:3 ratio when comparing bacterial and viral challenges.

Porcine reproductive and respiratory virus (PRRSV) is a pathogen that results in significant impacts on sow reproduction as well as respiratory disease in weaned and growing pigs. The economic losses due to PRRSV are estimated at $664 million each year for U.S. swine producers (Montaner-Tarbes et al., 2019). PRRSV is in the Arteriviridae family, order Nidovirales, and is an enveloped, positive-stranded RNA virus. The detection of North American and European PRRSV strains can be accomplished by using real-time PCR testing (Kleiboeker et al., 2005).

Lipopolysaccharide (LPS) is an acceptable challenge model to evaluate the immune system to study the response to Gram-negative bacterial infections (Wyns et al., 2015). When an LPS challenge is administered, macrophages and neutrophils produce and release cytokines. Pro-inflammatory cytokines, such as tumor necrosis factor-alpha (TNF-α), interleukin-1-beta (IL-1β), and interleukin-6 (IL-6), stimulate the acute phase immune response by activating hepatocytes to produce acute phase proteins. Pro-inflammatory cytokines also stimulate the hypothalamus-pituitary-adrenal axis, which produces prostaglandins, initiating the body to induce increased body temperatures. (Son et al., 2002; Llamas Mota et al., 2006).

O3 trial feed is a flax seed and Nannochloropsis algae-derived source of omega-3 fatty acids (alpha-linolenic acid) that has been used to increase omega-3 content of pork. The fatty acid profile makes it a viable option to reduce the omega-6:3 fatty acid ratio in nursery pig diets and potentially improve immune function. However, there is no published research available with O3 trial feed as a source of omega-3 fatty acids in nursery pigs. Our hypothesis was that lowering the ratio of dietary omega-6:3 fatty acids would enhance the pig’s immune system and reduce morbidity and mortality in light of an LPS or PRRSV challenge. Therefore, the objective of these studies was to determine the influence of omega-3 fatty acids (alpha-linolenic acid), sourced by O3 trial feed, on nursery pig growth performance, response to an LPS immune challenge, and morbidity and mortality in PRRSV-positive pigs in a commercial setting.

## Materials and Methods

### General

The Kansas State University Institutional Animal Care and Use Committee approved the protocol used in these experiments. In all three experiments, the omega-6:3 ratio was manipulated by adding a dry flaxseed and Nannochloropsis algae-based ingredient, rich in alpha-linolenic acid (O3 trial feed, NBO3 Technologies LLC, Manhattan, KS; Table 1) replacing corn on an equal weight basis. Feed-grade amino acids were adjusted slightly so that the amount of soybean meal was identical in all diets by phase.

**Table 1.** Analyzed fatty acid composition of O3 trial feed^1^

Fatty acid, %	Experiment 1	Experiment 2	Experiment 3
Total fatty acids	23.29	23.77	20.13
Total fat	25.87	26.41	22.37
Omega-6:3	0.36	0.37	0.46
C16:0	1.43	1.43	1.38
C18:1n9c	4.17	4.33	4.67
C18:2n6c^2^	4.28	4.41	4.03
C18:3n3^3^	12.09	12.20	8.73

^1^A representative sample of O3 trial feed was collected within each experiment and submitted to NBO3 Technologies LLC (Manhattan, KS) for fatty acid analysis.

^2^Major omega-6 fatty acid.

^3^Major omega-3 fatty acids.

### Experiment 1

Experiment 1 was conducted at the Kansas State University Swine Teaching and Research Center in Manhattan, KS. The facility is completely enclosed, environmentally controlled, and mechanically ventilated. Each pen contains a 4-hole, dry self-feeder, and a nipple waterer to provide ad libitum access to feed and water. Pens (1.2 × 1.2 m) had metal tri-bar floors and allowed ~0.288 m^2^/pig. A total of 350 weanling pigs (241 × 600, DNA, Columbus NE; initially 5.8 ± 0.03 kg) were used in a 41-d trial. There were five pigs per pen and 14 replications per treatment. Within experiments 1 and 2, pigs were placed in mixed-sex pens (experiments 1 and 2) or within room (experiment 3) with approximately the same number of barrows and gilts in each pen or room. Pens of pigs were randomly assigned to one of the five dietary treatments in a completely randomized design. The dietary treatments included increasing omega-3 fatty acids (0%, 1%, 2%, 3%, and 4% O3 trial feed) from weaning to day 41. Experimental diets were fed across three phases and were corn–soybean meal-based. Phase 1 diets were fed from days 0 to 13 (~5.8 to 7.3 kg BW). Phase 2 diets were fed from days 13 to 22 (~7.3 to 11.5 kg BW). Phase 3 was fed from days 22 to 41 (~11.5 to 22.8 kg BW). Diets were formulated to 1.40% SID Lys for phase 1, 1.35% SID Lys for phase 2, and 1.30% SID Lys for phase 3 (Table 2). All other nutrients were formulated to meet or exceed NRC (2012) requirement estimates. For phases 1 and 2, a single base diet was manufactured (Hubbard Feeds, Beloit, KS), then O3 trial feed, corn, and soybean meal additions were mixed at the Kansas State University O.H. Kruse Feed Technology Innovation Center (Manhattan, KS) to make the final diets. Complete phase 3 diets were manufactured at Hubbard Feeds (Beloit, KS). Phase 1 was fed in pellet form and phases 2 and 3 were fed in meal form. Individual pigs were weighed, and feed disappearance was recorded on days 0, 7, 13, 20, 22, 32, and 41 to determine ADG, ADFI, and G:F.

**Table 2.** Composition of experimental diets in experiment 1 (as-fed basis)^1^

Ingredient, %	Phase 1	Phase 2	Phase 3
Corn	40.32	56.37	66.12
Soybean meal (46.5% CP)	18.98	24.31	29.00
Dried whey	25.00	—	—
Whey permeate, 80% lactose	—	9.00	—
Corn DDGS^2^, 7.5% oil	5.00	—	—
Enzymatically treated soybean meal^3^	5.00	5.00	—
Corn oil	2.00	1.00	1.00
Calcium carbonate	0.50	0.75	0.75
Monocalcium P (21% P)	0.80	1.10	0.95
Sodium chloride	0.30	0.55	0.60
L-Lys-HCl	0.55	0.55	0.53
DL-Met	0.25	0.25	0.22
L-Thr	0.22	0.25	0.23
L-Trp	0.05	0.04	0.04
L-Val	0.17	0.17	0.16
Vitamin premix with phytase	0.25	0.25	0.25
Trace mineral premix	0.15	0.15	0.15
Choline chloride	0.04	—	—
Zinc oxide	0.41	0.25	—
Phytase^4^	0.02	0.02	0.02
O3 trial feed^5^	+/−	+/−	+/−
Calculated analysis			
Standardized ileal digestible (SID) AA, %			
Lys	1.40	1.35	1.30
Ile:Lys	55	54	54
Leu:Lys	110	108	114
Met and Cys:Lys	58	58	58
Thr:Lys	64	63	64
Trp:Lys	19.5	19.1	18.9
Val:Lys	70	70	71
His:Lys	32	34	36
Total Lys, %	1.53	1.48	1.44
NE, kcal/kg	2,588	2,522	2,489
SID Lys:NE, g/Mcal	5.41	5.35	5.22
CP, %	20.5	20.3	20.2
Ca, %	0.65	0.69	0.64
P, %	0.66	0.63	0.58
Standardized total tract digestible (STTD) P, %	0.58	0.51	0.46
Ca:P	0.99	1.10	1.10
EFA, %	2.34	1.91	2.06
Alpha-linolenic acid %	0.08	0.08	0.08
Linoleic acid %	2.26	1.83	1.98
Omega-6:3	27.3	23.0	24.4

^1^Phase 1 diets were fed from days 0 to 13 (~5.8 to 7.3 kg BW). Phase 2 diets were fed from days 13 to 22 (~7.3 to 11.5 kg BW). Phase 3 was fed from days 22 to 41 (~11.5 to 22.8 kg BW).

^2^Dried distillers grains with solubles.

^3^Hamlet Protein, Findlay, OH.

^4^Quantum Blue 5G (AB Vista, Marlborough, UK) provided a release of 0.13% STTD P with 411 FTU/kg.

^5^O3 trial feed was added at 0%, 1%, 2%, 3%, and 4% at the expense of corn from the experimental diets (NBO3 Technologies LLC, Manhattan, KS).

On day 25, two pigs per pen (those closest to the average weight of the pen) were injected intramuscularly in the neck with 20 µg *Escherichia coli* (*E. coli*) LPS per kg BW. An additional pig in each pen was injected with 2 mL of saline to serve as a control. The distribution of sex for pigs challenged with LPS and saline was approximately even. The LPS (*Escherichia coli* serotype O55:B5, Sigma Aldrich, Saint Louis, MO) was dissolved in sterile 0.9% NaCl solution. Body temperature was taken from all three pigs prior to the injection (0 h) and at 2, 4, 6, and 12 h after injection. A blood sample was taken from pigs injected with the *E. coli* LPS challenge one day prior to the challenge (day 24) and 4 h after the *E. coli* LPS injection to determine immune response criteria. Blood samples were centrifuged at 4 °C at 1,800 × *g* for 30 min and, then serum was frozen in separate aliquots for later cytokine analyses.

For IL-1β analysis, samples were analyzed in triplicate within a single assay. Serum concentrations of IL-1β were determined utilizing a porcine IL-1β ELISA kit per the instructions of the manufacturer (R & D Systems, Minneapolis, MN). The dynamic range of the assay was 39.1 to 2,500 pg/mL with a sensitivity of 13.6 pg/mL. For TNF-α analysis, samples were analyzed in triplicate within a single assay. Serum concentrations of TNF-α were determined using a porcine TNF-α ELISA kit per the instructions of the manufacturer (R & D Systems, Minneapolis, MN). The dynamic range of the assay was 23.4 to 1,500 pg/mL with a sensitivity of 5.0 pg/mL. Any sample with values outside the dynamic range of the assay was diluted and reanalyzed in triplicate.

### Experiment 2

This study was conducted at the New Fashion Pork Research Nursery in Jackson, MN. At weaning, pigs were moved to and housed in a temperature-controlled nursery facility. Each pen (1.89 × 3.05 m) consisted of plastic-grated flooring, one cup waterer, and one three-hole stainless steel self-feeder. Access to feed and water was provided ad libitum. Pigs were allowed ~0.262 m^2^/pig. A total of 1,056 weaned pigs [PIC TR4 × (Fast LW × PIC L02) initially 7.3 ± 0.09 kg] were used in a 46-d nursery trial. There were 22 pigs per pen (equal mixed sex) and 12 replications per treatment. Pens of pigs were randomly assigned to one of the four dietary treatments in a completely randomized design. The dietary treatments included increasing levels of omega-3 fatty acids (0%, 0.75%, 1.5%, and 3% O3 trial feed; Table 4). Experimental diets were fed across four phases and were corn–soybean meal based. Pigs were fed on a feed budget with phases 1, 2, 3, and 4 provided at 2.7, 3.8 7.3, and 14.5 kg per pig, respectively. Phases 1, 2, and 3 diets were formulated to 1.40% SID Lys and the phase 4 diet was formulated to 1.34% SID Lys. All other nutrients were formulated to meet or exceed NRC (2012) requirement estimates. All diets were manufactured at the New Fashion Pork Feed Mill (Estherville, IA) and fed in meal form. Pens of pigs were weighed and feed disappearance was recorded weekly during the course of this study to determine ADG, ADFI, and G:F. Cotton ropes were placed in each pen on days 7, 14, 21, 28, 35, and 42 to determine presence of the North American and European PRRSV strains in oral fluid samples. A new rope was placed in each pen for 15 min on each sample day and then oral fluids collected from six random pens were pooled for qRT-PCR testing. Samples were processed at the University of Minnesota Veterinary Diagnostic Laboratory using a commercially available qRT-PCR kit (Thermo-Fisher NA/EU PRRSV PCR; Waltham, MA).

### Experiment 3

This study was conducted at Seaboard Foods in northwest Oklahoma and southwest Kansas. At weaning, pigs were moved and housed in temperature-controlled nursery facilities. Each barn contained two rooms and each room contained 40 pens with 27 to 28 pigs per pen (~1,100 pigs per room). Each pen (2.97 × 1.97 m) contained one nipple waterer and one six-hole stainless steel self-feeder. Access to feed and water was provided ad libitum. Pigs were allowed ~0.201 m^2^/pig. A total of 91,140 weaned pigs (DNA 600 × PIC 1050; initially 5.1 ± 0.05 kg), originating from PRRSV-positive sow farms, were used across eight nursery sites. Each site contained five barns with two rooms in each barn. Rooms of pigs were blocked by nursery site and allocated within sow source, to one of the two dietary treatments with 40 groups (rooms) per treatment. The first treatment was a standard nursery diet program specific to the production system and did not contain O3 trial feed. The second treatment was the same standard nursery diet with 3% O3 trial feed. At placement, all pigs received 0.45 kg/pig of a common pre-starter diet containing no O3 trial feed. Pigs were then fed experimental diets across three phases (Table 6). Pigs were fed on a feed budget, receiving 2.7 kg/pig of phase 1 and 6.8 kg of phase 2 before being fed phase 3 for the remainder of the study. The SID Lys concentration was formulated to 1.35% for phase 1, 1.30% for phase 2, and 1.28% for phase 3. All other nutrients were formulated to meet or exceed NRC (2012) requirement estimates. All diets were corn–soybean meal-based and fed in pelleted form. O3 trial feed was added at the expense of corn while adjusting feed-grade amino acids and enzymatically treated soybean meal to maintain similar soybean meal levels and SID amino acid profiles. All diets contained a feed-grade antimicrobials. Diets for phases 1 and 2 were manufactured at Seaboard Feed Mill (Leoti, KS) and phase 3 was manufactured at Seaboard Feed Mill (Hugoton, KS). One truckload of weaned pigs was weighed each week. The truck weight was divided by the count of weaned pigs to create a weekly initial BW for pigs placed in the nursery. At the end of each nursery turn, pigs were weighed by truckloads to determine close-out weights for each room. Feed intake was determined by the difference between the amount of feed delivered and the feed remaining upon completion of the nursery group. These data were used to determine ADG, ADFI, and G:F. Adjusted ADG was calculated by adding total removal and mortality weight to the total gain to calculate adjusted total gain, which then was divided by pig days. After the shipping of each nursery room, all water and injectable treatment records were collected. Cotton ropes were placed in each room every other week to evaluate for the presence of the North American and European PRRSV strains in oral fluid samples. New ropes were placed in each room for 30 min on each sample day and then oral fluids collected from each rope were pooled to create two duplicate samples for each room. The oral fluid samples were then frozen and sent to Kansas State University Swine Lab and stored at −20 °C. Samples were processed at Kansas State University Veterinary Diagnostic Laboratory using the Tetracore PRRS Multiplex real-time PCR procedure.

### Chemical Analysis

In experiment 1, phases 1 and 2 diet samples were collected at manufacturing, and phase 3 diet samples were collected from every fifth 22.7-kg bag using a feed probe to obtain a representative sample for each respective diet and phase. In experiment 2, diet samples for each treatment were collected with a probe from feeders. Complete diet samples were stored at −20 °C until they were homogenized, subsampled, and submitted for analysis. In experiment 3, diet samples for each treatment were collected weekly from feeders in each room throughout the study. Complete diet samples were sent to Kansas State University Swine Lab and stored at −20 °C. Samples were then subsampled to create a composite sample for each treatment and submitted for analysis. Samples of each dietary treatment were analyzed (NBO3 Technologies LLC; Manhattan, KS) for fatty acid profiles (Tables 3, 5, and 7). Also, a representative sample of O3 trial feed was collected within each experiment and submitted to NBO3 Technologies LLC (Manhattan, KS) for fatty acid analysis (Table 1).

**Table 3.** Analyzed fatty acid composition of experimental diets in experiment 1^1^

	O3 trial feed, %				
Fatty acid, %	0	1	2	3	4
Phase 1 (days 0 to 13)					
Total fatty acids	4.21	4.34	4.72	4.96	4.89
Total fat	4.68	4.82	5.25	5.51	5.44
Omega-6:3	18.57	9.56	6.44	4.94	4.07
C16:0	0.61	0.61	0.64	0.66	0.64
C18:1n9c	1.01	1.01	1.10	1.15	1.12
C18:2n6c^2^	2.26	2.25	2.36	2.11	2.32
C18:3n3^3^	0.12	0.24	0.37	0.49	0.57
Phase 2 (days 13 to 22)					
Total fatty acids	3.95	3.97	4.64	4.53	4.56
Total fat	4.39	4.41	5.15	5.04	5.06
Omega-6:3	15.03	9.60	5.38	4.29	3.78
C16:0	0.57	0.56	0.61	0.59	0.58
C18:1n9c	0.89	0.88	1.03	0.98	0.98
C18:2n6c	2.14	2.11	2.31	2.21	2.17
C18:3n3	0.14	0.22	0.43	0.52	0.58
Phase 3 (days 22 to 41)					
Total fatty acids	4.47	4.77	5.01	4.49	4.71
Total fat	4.96	5.30	5.61	4.99	5.23
Omega-6:3	20.69	10.11	6.71	4.86	3.80
C16:0	0.66	0.67	0.68	0.61	0.61
C18:1n9c	1.06	1.11	1.18	0.99	1.03
C18:2n6c	2.36	2.46	2.53	2.17	2.20
C18:3n3	0.11	0.24	0.38	0.45	0.58

^1^Complete diets contained trace levels of C12:0, C14:0, C15:0, C16:1n7, C17:0, C18:0, C18:1n9t, C18:1n7c, C18:3n6, CLA 9c, 11t (n7), C20:0, C20:2n6, C22:0, C23:0, and C24:0 of < 0.10%. Other fatty acid levels were too low to be detected in the analysis.

^2^Major omega-6 fatty acids.

^3^Major omega-3 fatty acids.

**Table 4.** Composition of experimental diets in experiment 2 (as-fed basis)^1^

Ingredient, %	Phase 1	Phase 2	Phase 3	Phase 4
Corn	42.46	55.59	52.97	43.46
Soybean meal (46.5% CP)	18.99	19.99	26.75	26.52
AV-E digest^2^	17.45	16.05	3.90	—
Dried whey	2.25	—	—	—
Oat groats	5.00	—	—	—
Cereal blend^3^	7.95	3.00	1.40	—
Corn DDGS^4^, 7.5% Oil	—	—	10.00	25.00
Beef tallow	2.50	2.00	1.00	1.00
Monocalcium P (21% P)	—	—	0.40	0.38
Limestone	0.40	0.40	1.15	1.48
Salt	—	0.16	0.55	0.63
L-Lys-HCl	0.45	0.49	0.58	0.56
DL-Met	0.19	0.22	0.32	0.22
L-Trp	0.05	0.06	0.05	0.04
L-Val	0.11	0.14	0.15	0.06
L-Ile	0.03	0.05	0.06	—
L- Thr	0.22	0.26	0.29	0.20
Tribasic copper chloride	0.04	0.04	—	—
Vitamin premix with phytase	0.25	0.25	0.25	0.25
Trace mineral premix	0.15	0.15	0.15	0.15
Choline chloride 60%	0.03	0.03	—	—
Zinc oxide	0.35	0.35	0.02	—
Vitamin E (20,000 IU)	0.05	0.01	0.01	—
Zinc^5^	—	—	—	0.06
LipoVital GL 90^6^	0.10	0.05	—	—
Blue dye	0.01	—	—	—
FXP^7^	0.20	0.10	—	—
AcidoMatrix GH^8^	0.50	0.50	—	—
N-Hance^7^	0.25	0.10	—	—
AlphaGal 280P^9^	0.03	0.01	—	—
Manganese^10^	0.02	0.02	0.02	0.01
O3 Trial Feed^11^	—	—	—	—
Calculated analysis				
Standardized ileal digestible (SID) amino acids, %				
Lys	1.40	1.40	1.40	1.34
Ile:Lys	59	59	60	60
Leu:Lys	111	113	120	141
Met and Cys:Lys	57	59	66	65
Thr:Lys	63	64	64	63
Trp:Lys	19.3	19.1	19.1	19.2
Val:Lys	71	72	72	72
His:Lys	36	36	36	40
Total Lys, %	1.62	1.62	1.58	1.53
NE NRC, kcal/kg	2,255	2,321	2,396	2,412
SID Lys:NE, g/Mcal	6.21	6.04	5.85	5.56
CP, %	23.9	23.5	23.1	24.1
Ca, %	0.87	0.80	0.79	0.80
P, %	0.61	0.58	0.56	0.57
Standardized total tract digestible (STTD) P, %	0.49	0.47	0.44	0.44
Ca:P	1.42	1.38	1.41	1.41
EFA, %	1.67	1.86	1.84	2.17
Alpha-linolenic acid %	0.11	0.11	0.08	0.08
Linoleic acid %	1.6	1.75	1.75	2.08
Omega-6:3	15.1	16.5	20.8	25.3

^1^Pigs were fed on a feed budget (kg/pig): phase 1, 2.7; phase 2, 3.6; phase 3, 7.3; and phase 4, 14.5 lb per pig.

^2^XFE Products, Des Moines, IA.

^3^Quincy Farm Products, Quincy, IL.

^4^Dried distillers grains with solubles.

^5^New Fashion Pork Custom Zinc: 210,000 ppm.

^6^Berg and Schmidt Functional Lipids, Hamburg, Germany.

^7^Ani-Tek, Social Circle, GA.

^8^Novus International, Saint Charles, MO.

^9^Kindstrom-Schmoll Inc., Eden Prairie, MN.

^10^New Fashion Pork Custom Manganese—200,000 ppm.

^11^O3 trial feed was added at 0.75%, 1.5%, and 3% at the expense of corn and soybean meal to form the experimental diets.

**Table 5.** Analyzed fatty acid composition of experimental diets in experiment 2^1^

	O3 trial feed, %			
Fatty acid, %	0.00	0.75	1.50	3.00
Phase 1				
Total fatty acid	7.21	8.36	8.17	8.86
Total fat	8.01	9.28	9.07	9.84
Omega-6:3	14.34	10.23	7.65	4.05
C16:0	1.38	1.60	1.51	1.62
C18:1n9c	2.16	2.56	2.36	2.63
C18:2n6c^2^	2.02	2.17	2.22	2.17
C18:3n3^3^	0.14	0.22	0.29	0.54
Phase 2				
Total fatty acid	6.54	6.96	7.92	8.25
Total fat	7.27	7.74	8.80	9.16
Omega-6:3	14.36	9.94	6.44	4.18
C16:0	1.23	1.29	1.45	1.47
C18:1n9c	1.93	2.06	2.31	2.39
C18:2n6c	1.95	2.03	2.24	2.27
C18:3n3	0.14	0.21	0.36	0.55
Phase 3				
Total fatty acid	5.48	5.48	5.60	6.39
Total fat	6.08	6.09	6.23	7.10
Omega-6:3	14.45	8.85	6.46	4.10
C16:0	0.89	0.87	0.88	0.96
C18:1n9c	1.34	1.32	1.31	1.52
C18:2n6c	2.32	2.27	2.31	2.49
C18:3n3	0.16	0.26	0.36	0.61
Phase 4				
Total fatty acid	5.65	5.91	6.15	6.48
Total fat	6.28	6.57	6.83	7.20
Omega-6:3	15.34	10.16	7.53	5.53
C16:0	0.90	0.90	0.93	0.97
C18:1n9c	1.35	1.40	1.46	1.53
C18:2n6c	2.56	2.64	2.68	2.73
C18:3n3	0.17	0.26	0.36	0.50

^1^Complete diets contained trace levels of C12:0, C14:0, C15:0, C16:1n7, C17:0, C18:0, C18:1n9t, C18:1n7c, C18:3n6, CLA 9c, 11t (n7), C20:0, C20:2n6, C22:0, C23:0, and C24:0 of < 0.10%. Other fatty acid levels were too low to be detected in the analysis.

^2^Major omega-6 fatty acids.

^3^Major omega-3 fatty acids.

**Table 6.** Composition of experimental diets in experiment 3 (as-fed basis)^1^

	Phase 1		Phase 2		Phase 3	
	O3 trial feed, %		O3 trial feed, %		O3 trial feed, %	
Ingredient, %	0	3	0	3	0	3
Corn	43.97	40.58	59.30	56.13	56.72	53.30
Soybean meal (47%)	20.00	20.00	32.50	32.50	37.25	37.83
Base mix^2^	21.13	21.13	—	—	—	—
Enzymatically treated soybean meal^3^	6.09	6.42	0.78	1.09	—	—
Lucrafit TM 50^4^	2.15	2.15	1.25	1.25	—	—
Monocalcium phosphate^5^	1.52	1.58	1.15	1.17	0.84	0.86
Beef tallow	0.86	0.86	1.64	1.54	2.48	2.39
Salt	0.73	0.73	0.60	0.60	0.40	0.40
Limestone, ground	0.41	0.40	0.54	0.54	0.66	0.66
L-Lys	0.68	0.64	0.65	0.61	0.46	0.41
L-Trp	0.10	0.10	0.10	0.09	0.08	0.07
L-Val	0.06	0.10	0.09	0.10	—	—
L-Thr	0.20	0.20	0.22	0.20	0.17	0.15
Vitamin premix-nursery^6^	0.05	0.05	0.05	0.05	—	—
Vitamin premix-grow-finish^7^	—	—	—	—	0.08	0.08
Trace mineral premix^8^	0.10	0.10	0.08	0.08	0.08	0.08
Phytase^9^	0.06	0.06	0.06	0.06	0.06	0.06
Copper chloride	0.03	0.03	0.03	0.03	0.03	0.03
Choline chloride	0.03	0.03	—	—	—	—
Zinc oxide 72%	0.32	0.32	0.32	0.32	—	—
FXP^10^	0.40	0.40	0.20	0.20	—	—
Liquid methionine^11^	0.30	0.31	0.21	0.21	0.17	0.15
N-hance^10^	0.30	0.30	—	—	—	—
CTC 100g	0.25	0.25	—	—	0.25	0.25
Oxytetracycline 200 g	—	—	0.13	0.13	—	—
Tiamulin 10 g	0.18	0.18	—	—	0.18	0.18
Synthetic red dye	—	0.01	—	0.01	—	0.01
Synthetic blue dye	0.01	—	0.01	—	0.01	—
O3 trial feed^12^	—	3.00	—	3.00	—	3.00
Calculated analysis						
Standardized ileal digestible (SID) amino acids, %						
Lys	1.35	1.35	1.30	1.30	1.28	1.28
Ile:Lys	58	58	58	58	64	64
Met and Cys:Lys	58	58	58	58	58	58
Thr:Lys	64	64	64	64	64	64
Trp:Lys	24	24	24	24	24	24
Val:Lys	72	72	72	72	70	70
Total Lys, %	1.46	1.46	1.43	1.43	1.42	1.43
NE NRC, kcal/kg	2,630	2,620	2,680	2,680	2,770	2,770
SID Lys:NE, g/Mcal	5.15	5.19	4.83	4.83	4.61	4.61
CP, %	21.79	22.68	21.51	22.00	22.58	23.14
Crude fat, %	4.10	4.42	4.10	4.52	4.80	5.22
Ca, %	0.68	0.68	0.67	0.67	0.70	0.70
P, %	0.67	0.68	0.59	0.59	0.54	0.54
Standardized total tract digestible (STTD) P, %	0.50	0.50	0.47	0.47	0.43	0.43

^1^Pigs were fed experimental diets on a feed budget with phases 1 and 2 provided at 2.7 and 6.8 kg per pig. Phase 3 was provided for the remainder of the study.

^2^Quincy Farms, Quincy IL.

^3^HP300; Hamlet Protein, Findley, OH.

^4^Purina Animal Nutrition, Arden Hills, MN.

^5^NexFos; The Mosaic Company, Plymouth, MN.

^6^Provided per kg of premix: 24,250,869 IU vitamin A; 3,747,862 IU vitamin D_3_; 220,462 IU vitamin E; 6614 mg menadione; 19,842 mg riboflavin; 97,003 mg niacin; 79,366 mg pantothenic acid; 93 mg vitamin B_12_; 220 mg biotin; 3,527 mg folic acid; 6,614 mg pyridoxine; 600 mg selenium; and 32,099,551 BXU xylanase.

^7^Provided per kg of premix: 8,818,498 IU vitamin A; 1,543,237 IU vitamin D_3_; 44,092 IU vitamin E; 3,307 mg menadione; 6,614 mg riboflavin; 27,558 mg niacin; 26,455 mg pantothenic acid; 26 mg vitamin B_12_; 375 mg selenium; and 25,623,400 BXU xylanase.

^8^Provided per kg of premix: 187,500 mg Zn from zinc oxide and zinc sulfate, 95,000 mg Fe from ferrous sulfate, 31,250 mg Mn from manganese sulfate and manganese oxide, 18,750 mg Cu from copper sulfate, and 750 mg I from calcium iodate.

^9^Axtra PHY Gold (Danisco Animal Nutrition, Cedar Rapids, IA) was included to provide ~1,730 FTU/kg in phase1, 1,875 FTU/kg in phase 2, and 1,920 FTU/kg in phase 3 providing an estimated release of 0.08, 0.11, and 0.12% STTD P, for phase 1, 2, and 3, respectively.

^10^Ani-Tek, Social Circle, GA.

^11^Alimet; Novus International Inc., Saint Charles, MS.

^12^O3 trial feed (NBO3 Technologies LLC, Manhattan, KS) was added at 3% at the expense of corn while adjusting feed grade amino acids and enzymatically treated soybean meal to maintain similar soybean meal levels and amino acid profiles.

**Table 7.** Analyzed fatty acid composition of experimental diets in experiment 3^1^

	O3 trial feed, %	
Fatty acid, %	0	3
Total fatty acid	5.14	5.44
Total fat	5.72	6.05
Omega-6:3	14.74	4.57
C16:0	0.99	0.95
C18:1n9c	1.41	1.44
C18:2n6c^2^	1.67	1.79
C18:3n3^3^	0.11	0.39

^1^Composites of complete diets contained trace levels of C6:0, C8:0, C10:0, C12:0, C14:0, C14:1n5, C15:0, C16:1n7, C17:0, C18:0, C18:1n9t, C18:1n7t, C18:1n7c, C18:3n6, CLA 9c, 11t (n7), C20:0, C201n9, C20:2n6, C22:0, C23:0, C24:0, and C24:1n9 of < 0.10%. Other fatty acids levels were too low to be detected in the analysis.

^2^Major omega-6 fatty acid.

^3^Major omega-3 fatty acid.

### Statistical Analysis

Growth performance (experiments 1 and 2) and mortality (experiment 2 only) data were analyzed as a completely randomized design with pen serving as the experimental unit. Linear and quadratic contrasts in response to increasing omega-3 fatty acids (increasing O3 trial feed), were measured among treatments. In experiment 3, growth performance data were analyzed as randomized complete block design with room serving as the experimental unit. Treatment was included in the model as a fixed effect, with nursery site and calendar week nested within sow farm origin included in the model as random intercepts. Models were fit with the nlme package of R (Version 4.0.0, R Foundation for Statistical Computing, Vienna, Austria). Mortality and medication data were analyzed using the GLIMMIX procedure of SAS (version 9.4, Cary, NC). Total removals and mortality data were analyzed assuming a binomial distribution with a logit link function. Medication data were analyzed using a Poisson distribution with an offset function using the log-transformed number of days at risk for each experimental unit or count of pigs placed and data reported as count of injections per 1,000 pig days and count of injections per pig placed, respectively. In experiment 1, cytokine and temperature data were analyzed using the lmer package of R. Treatment, time, and the associated interaction were included in the model as fixed effects to account for repeated measurements over time, with random effects of pen and plate. Values were calculated by subtracting the measured variable at each time point from the baseline level (h 0). Pearson correlation coefficients (*r*) were determined using PerformanceAnalytics package of R. The coefficient of determination (*R*^2^) is calculated by squaring the Pearson correlation coefficient. Differences between treatments were considered significant at *P* ≤ 0.05 and marginally significant at 0.05 < *P *≤ 0.10.

## Results

### Chemical Analysis

For all three experiments, fatty acid analysis in treatment diets were similar to formulated values. As the O3 trial feed increased in the diet, the level of omega-3 fatty acids increased and the omega-6:3 fatty acid ratio decreased (Tables 3, 5, and 7).

### Experiment 1

In phase 1 (days 0 to 13), there was a tendency (linear, *P* = 0.065; Table 8) for increased ADG with increasing O3 trial feed. For ADFI, there was a quadratic (*P* = 0.046) effect with ADFI decreasing as O3 trial feed increased from 0% to 1% and then increasing as O3 trial feed increased up to 4%. There were no significant differences observed for G:F. On day 13, due to the numerical increase in ADG, increasing O3 trial feed increased BW (linear, *P* = 0.042). In phase 2 (days 13 to 22), there were no differences observed in ADG or G:F. However, increasing O3 trial feed increased ADFI as O3 trial feed increased up to 2% (quadratic, *P* = 0.013), with ADFI decreasing as O3 trial feed increased. In phase 3 (days 22 to 41), no differences were observed in ADG or ADFI. However, during this period, increasing O3 trial feed improved (linear, *P* = 0.046) G:F. For overall growth performance (days 0 to 41), there were no significant differences observed in ADG, ADFI, G:F, or final BW.

**Table 8.** Effects of omega-3 fatty acids, sourced by O3 trial feed, on nursery pig performance and cytokine production in experiment 1^1^

	O3 trial feed,^2^ %						P =	
Item	0	1	2	3	4	SEM	Linear	Quadratic
BW, kg								
day 0	5.8	5.7	5.8	5.8	8.8	0.03	0.366	0.927
day 13	7.2	7.0	7.4	7.3	7.4	0.12	0.042	0.831
day 22	11.6	11.1	11.8	11.8	11.4	0.18	0.361	0.441
day 41	23.0	21.8	23.3	22.9	22.7	0.32	0.534	0.975
Phase 1 (days 0 to 13)								
ADG, g	110	97	126	112	126	8.08	0.065	0.732
ADFI, g	196	174	199	205	237	12.01	0.003	0.046
G:F, g/kg	556	551	630	556	554	34.3	0.993	0.245
Phase 2 (days 13 to 22)								
ADG, g	480	447	482	489	446	12.0	0.502	0.281
ADFI, g	600	608	633	621	576	15.6	0.487	0.013
G:F, g/kg	802	739	763	791	774	16.1	0.947	0.116
Phase 3 (days 22 to 41)^3^								
ADG, g	602	567	606	585	595	12.5	0.914	0.544
ADFI, g	954	950	940	916	926	21.8	0.188	0.869
G:F, g/kg	631	602	645	639	643	9.9	0.046	0.646
Overall (days 0 to 41)								
ADG, g	415	384	425	408	411	7.8	0.497	0.695
ADFI, g	630	615	634	616	627	11.3	0.889	0.726
G:F, g/kg	659	625	670	662	657	9.5	0.264	0.906
IL-1β change, pg/mL^4^	506.1	615.5	777.8	430.6	543.2	147.8	0.796	0.308
TNF-α change, pg/mL^5^	5,002	6,093	5691	5,628	4,463	1,001.8	0.623	0.272

^1^A total of 350 pigs (line 241 × 600, DNA, Columbus NE; initially 5.8 ± 0.03 kg) were used with five pigs per pen and 14 replications per treatment and were fed trial diets for a 41-d period.

^2^Omega-6:3 ratios for the five treatments within each phase were: phase 1 (27.3:1, 11.6:1, 7.4:1, 5.4:1, and 4.3:1); phase 2 (23.0:1, 9.6:1, 6.1:1, 4.5:1, and 3.6:1); and phase 3 (24.4:1, 10.1:1, 6.5:1, 4.8:1, and 3.8:1), respectively (NBO3 Technologies LLC, Manhattan, KS).

^3^Two pigs per pen were injected intramuscularly with 20 micrograms *Escherichia coli* (*E. coli*) LPS per kg BW on day 25 to measure immune responses.

^4^Change in IL-1β from baseline (0 h) to 4 h after intramuscular injection with 20 µg *Escherichia coli* LPS per kg BW on day 25. The average IL-1β baseline was 4.1 ± 1.68 pg/mL across all treatments and with all baseline values below 21.5 pg/mL.

^5^Change in TNF-α from baseline (0 h) to 4 h after intramuscular injection with 20 µg *Escherichia coli* LPS per kg BW on d 25. The average TNF-α baseline was 114.5 ± 0.24 pg/mL across all treatments and with all baseline values below 527.7 pg/mL.

**Figure 1.**
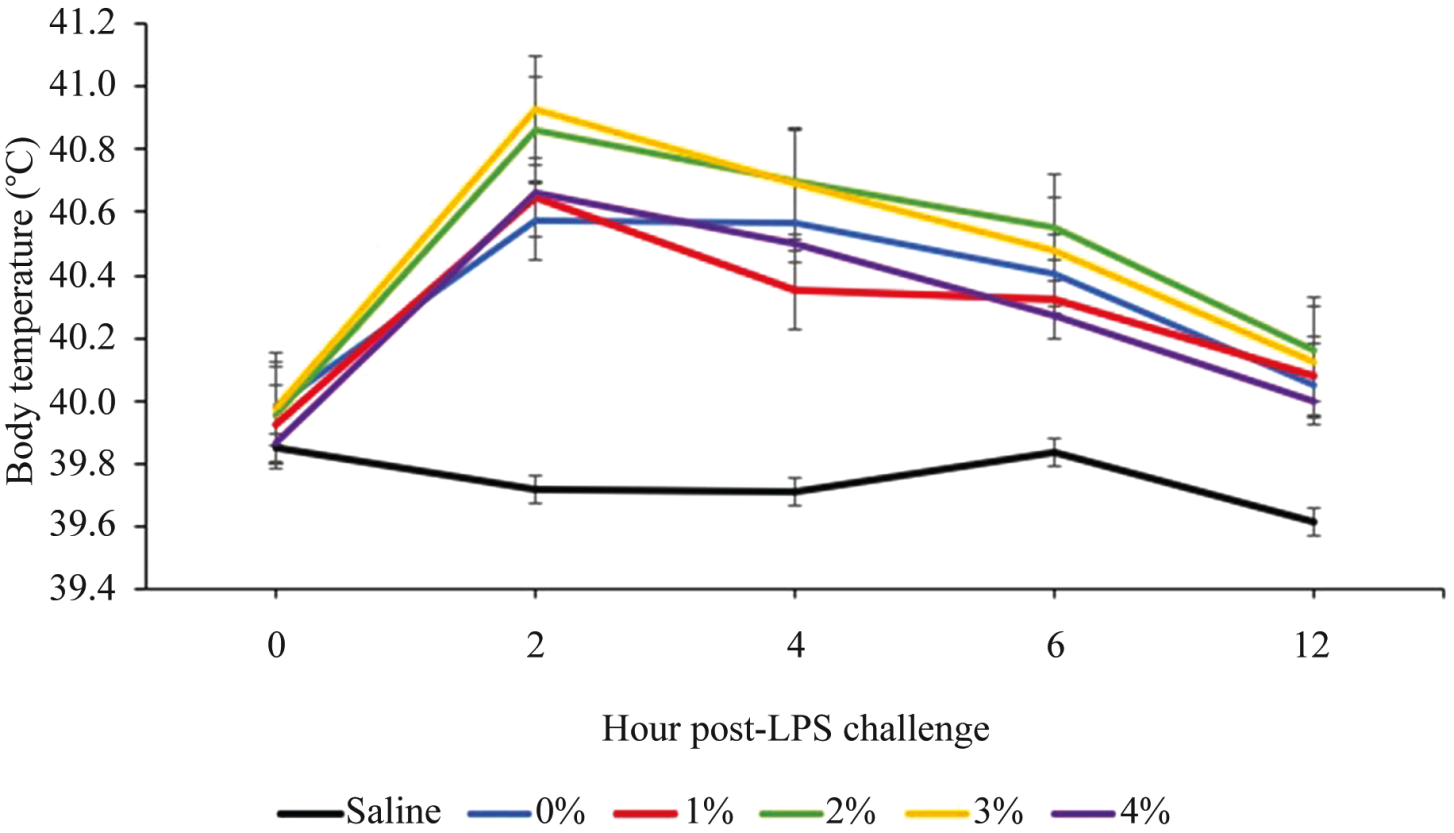
Body temperature (°C) over time between treatments. There was no treatment × time interaction (*P* > 0.10) in body temperatures over time. There was also no main effect of treatment (*P* > 0.10). There was a main effect of time (*P* < 0.001).

Prior to the LPS challenge (h 0), the average pig body temperature was 39.9 ± 0.28 °C. There was no interaction between treatment and time in change in body temperature with increasing O3 trial feed, nor main effect of O3 trial feed on temperature (*P* > 0.10; Figure 1). However, there was a main effect of time (*P* < 0.001). Pigs responded as expected with an increase in body temperature at 2 h post-LPS challenge with average body temperature 2 h post-LPS challenge being 40.4 ± 0.65 °C. Then, body temperature decreased as time post-challenge increased. After 12 h post-LPS challenge, body temperatures were close to baseline levels with an average body temperature of 39.9 ± 0.45 °C. Increasing O3 trial feed did not influence IL-1β or TNF-α concentrations from baseline to 4 h post LPS challenge (*P* > 0.10). The average IL-1β baseline was 4.1 ± 1.68 pg/mL across all treatments with all values below 21.5 pg/mL. The average TNF-α baseline was 114.5 ± 0.24 pg/mL across all treatments with all values below 527.7 pg/mL.

### Experiment 2

The North American strain of PRRSV was not detected in oral fluids taken from ropes that were placed in pens on days 7 and 14, but was detected on days 21, 28, 35, and 42. The European strain of PRRSV was undetectable on each collection day.

From days 0 to 14 and days 14 to 21, there were no differences observed for ADG or ADFI (*P* > 0.10; Table 9). However, from days 0 to 14, increasing O3 trial feed resulted in a tendency for an increase and then decrease (quadratic, *P* = 0.065) in G:F with G:F increasing through 1.5% and decreasing thereafter. From days 21 to 28 corresponding with the detection of PRRSV in the population, pigs fed increasing O3 trial feed had increased (linear, *P* ≤ 0.035) ADG and ADFI and improved (linear, *P* = 0.010) G:F. From days 28 to 35, ADG (quadratic, *P* = 0.009) and G:F (quadratic, *P* = 0.004) decreased with increasing O3 trial feed, but then returned to control values at 3% O3 trial feed. During this period, increasing O3 trial feed increased (linear, *P* = 0.016) ADFI. Finally, from days 35 to 46, increasing O3 trial feed increased (linear, *P *≤ 0.001) ADG, ADFI, and G:F (linear, *P* = 0.010).

**Table 9.** Effects of omega-3 fatty acids, sourced by O3 trial feed on growth performance and total removals and mortality in PRRSV-challenged nursery pigs in experiment 2^1^

Item	O3 trial feed,^2^ %					P =	
	0.00	0.75	1.50	3.00	SEM	Linear	Quadratic
BW, kg							
day 0	7.3	7.3	7.3	7.3	0.09	0.315	0.601
day 14	8.9	8.9	9.1	8.9	0.09	0.735	0.138
day 21	11.4	11.3	11.4	11.3	0.12	0.916	0.980
day 28	13.5	13.6	13.6	14.0	0.17	0.023	0.528
day 35	17.8	17.6	17.9	18.5	0.21	0.006	0.169
day 46	22.4	22.9	23.2	24.0	0.24	<0.001	0.987
days 0 to 14							
ADG, g	99	100	117	108	6.2	0.139	0.142
ADFI, g	175	174	178	178	5.3	0.508	0.870
G:F, g/kg	562	565	653	604	22.8	0.090	0.065
days 14 to 21							
ADG, g	343	337	323	345	9.3	0.926	0.108
ADFI, g	514	494	493	492	10.2	0.184	0.268
G:F, g/kg	668	683	656	702	17.5	0.233	0.339
days 21 to 28							
ADG, g	303	324	319	380	12.9	<0.001	0.285
ADFI, g	569	604	601	617	13.9	0.035	0.379
G:F, g/kg	539	534	530	615	21.2	0.010	0.079
days 28 to 35							
ADG, g	615	578	594	635	12.1	0.062	0.009
ADFI, g	797	788	903	837	13.1	0.016	0.251
G:F, g/kg	771	734	740	758	8.6	0.732	0.004
days 35 to 46							
ADG, g	411	458	464	493	12.8	<0.001	0.218
ADFI, g	801	826	842	883	17.1	0.001	0.936
G:F, g/kg	513	554	551	560	10.3	0.010	0.077
days 0 to 46 (Overall)							
ADG, g	315	322	331	355	5.3	<0.001	0.568
ADFI, g	522	528	539	557	7.5	<0.001	0.844
G:F, g/kg	603	611	635	637	5.3	<0.001	0.510
Total removals and mortalities, %	11.5	11.9	8.5	6.7	2.14	0.027	0.856

^1^A total of 1,056 pigs (PIC TR4 × (Fast LW × PIC L02) initially 7.3 ± 0.09 kg) were used with 22 pigs per pen and 12 replications per treatment and were fed trial diets for a 46-d period.

^2^Omega-6:3 ratios for the four treatments within each phase were: Phase 1 (15.1:1, 8.4:1, 5.9:1, and 3.7:1); phase 2 (16.5:1, 9.2:1, 6.4:1, and 4.0:1); phase 3 (20.8:1, 10.4:1, 7.0:1, and 4.2:1); and phase 4 (25.3:1, 12.5:1, 8:3:1, and5.0:1), respectively (NBO3 Technologies LLC, Manhattan, KS).

For overall growth performance (days 0 to 46), increasing O3 trial feed increased (linear, *P* < 0.001) ADG, resulting in pigs fed 3% O3 trial feed having the greatest growth rate. Increasing O3 trial feed also increased (linear, *P* < 0.001) overall ADFI and G:F. Percentages of total removals and mortality for the overall study decreased (linear, *P* = 0.027) with increasing O3 trial feed.

### Experiment 3

Of the oral fluids taken from the ropes placed in each room, the North American strain of PRRSV was detected in 61 of the 80 rooms (76%) at the first sampling period 1-wk post-placement in the nursery. The North American strain of PRRSV was detected in 78 of the 80 rooms (98%) tested at the second sampling point, 3 wk post-placement in the nursery, and all oral fluid samples from each room had detectable North American PRRSV for the remainder of the nursery turn. The European strain of PRRSV was not detected in any sample.

Overall, there were no differences observed in ADG, ADFI, or G:F between pigs fed the control diet or those fed 3% O3 trial feed (*P* > 0.10; Table 10). Similarly, there were no differences observed for adjusted ADG or G:F (*P* > 0.10). There were also no main effects of sow flow between pigs fed the control diets or those fed diets containing 3% O3 trial feed (*P* > 0.10). Pigs fed control diets had reduced (*P* < 0.001) total removals and mortalities compared to pigs fed diets containing 3% O3 trial feed.

**Table 10.** Effects of omega-3 fatty acids, sourced by O3 trial feed on growth performance, total removals and mortality, and medication usage in PRRSV-challenged nursery pigs in experiment 3^1^

	O3 trial feed, %			
Item	0	3	SEM	*P* =
BW, kg				
days 0	5.2	5.1	0.05	0.316
day 43	17.7	17.6	0.31	0.707
Overall (days 0 to 43)				
ADG, g	285	281	7.0	0.555
ADFI, g	434	426	12.5	0.313
G:F, g/kg	662	663	22.8	0.924
Total removals and mortality, %	7.7	8.9	1.13	<0.001
Total injections				
Injections per 1,000 pig days, n	18.26	18.03	1.170	0.226
Injections per pig placed, *n*	0.70	0.68	0.042	<0.001

^1^A total of 91,140 (initially 5.1 ± 0.05 kg) were used with 40 rooms per treatment and ~1,100 pigs per room.

Pigs fed diets containing 3% O3 trial feed had fewer (*P* < 0.001; Table 10) total injections per pigs placed compared to pigs fed diets without O3 trial feed. However, there were no significant differences observed in the total number of injections given per 1,000 pig days (*P* > 0.10). Of the medication administered, 95%–97% was enrofloxacin with the remainder of the medication given being dexamethasone, and ceftiofur hydrochloride.

Data were analyzed to understand the potential correlation between PRRSV status and growth performance. A higher cycle threshold (Ct) value means that there is less detectable viral genomic material present. In contrast, a lower Ct value means that there is more viral genomic material present in the sample. For oral fluids collected 1-wk post-placement, ADG was significantly and positively correlated with Ct values (*P* < 0.01; *r* = 0.44; Table 11). This shows that 19% (*R*^2^) of the variability in ADG is explained by the Ct value 1-wk post-placement. Similarly, there was a significant and positive correlation between ADFI and Ct values for oral fluids collected 1-wk post-placement (*P* < 0.05; *r* = 0.33). This explains that 11% of the variability in ADFI is described by Ct values 1-wk post-placement. However, there was no correlation between G:F and Ct values (*P *> 0.10). These results show that decreased Ct values at placement (greater amount of viral RNA) are associated with reductions in ADG and ADFI. There was a significant and negative correlation between total removals and mortality and Ct values for oral fluids collected 1-wk post-placement (*P* < 0.01; *r* = −0.67), and a significant negative correlation between total removals and mortality and Ct values for oral fluids collected 3-wk post-placement (*P* < 0.05; *r* = −0.30). This shows that 45% and 9% of the variability in total removals and mortality is explained by Ct values 1- and 3-wk post-placement, respectively. There was no evidence of correlation between oral fluid samples collected late in the nursery phase with total removals and mortality (*P* > 0.10).

**Table 11.** Correlations between Ct values, growth performance, and removals and mortality in experiment 3^1^

	Cycle threshold (Ct) value			
	Week 1	Week 3	Week 5	Week 7
ADG, g	*r* = 0.44	*r* = 0.26	*r* = 0.04	*r* = −0.07
	*P* < 0.01	*P* < 0.10	*P* > 0.10	*P* > 0.10
ADFI, g	*r* = 0.33	*r* = 0.18	*r* = 0.10	*r* = 0.17
	*P* < 0.05	*P* > 0.10	*P* > 0.10	*P* > 0.10
G:F, g/kg	*r* = 0.18	*r* = 0.11	*r* = −0.04	*r* = −0.18
	*P* > 0.10	*P* > 0.10	*P* > 0.10	*P* > 0.10
Removals and mortality, %	*r* = −0.67	*r* = −0.30	*r* = 0.07	*r* = 0.09
	*P* < 0.01	*P* < 0.05	*P* > 0.10	*P* > 0.10

^1^A total of 91,140 (Initially 5.1 ± 0.05 kg) were used with 40 rooms per treatment and ~1,100 pigs per room.

## Discussion

As productivity in the swine industry increases, so does prevalence of bacterial and viral diseases (Davies, 2011). Thus, it is important to continue to improve the interaction between health and nutrition to maximize production efficiency and immune function (Carroll et al., 2003). One nutritional strategy to potentially influence the immune response is to incorporate omega-3 fatty acids (alpha-linolenic acid) into nursery diets.

Polyunsaturated fatty acids (PUFA) are categorized into two classes: omega-6 and omega-3 fatty acids. Omega-6 fatty acids are derived from linoleic acid and omega-3 fatty acids are derived from alpha-linolenic acid. Both fatty acids cannot be synthesized in the body; therefore, they must be added to the diet. Omega-3 fatty acids are converted into eicosatetraenoic acid (EPA) and later converted into docosahexaenoic acid (DHA; Teitelbaum and Walker, 2001). Omega-6 fatty acids are converted into arachidonic acid. Typical corn–soybean meal-based swine diets contain high levels of arachidonic acid and low levels of EPA and DHA (Liu, 2015). The addition of omega-3 fatty acids to the diet at the expense of omega-6 fatty acids increases the incorporation of EPA and DHA into the phospholipid layer of cells which can play a role in the regulation of inflammation and immune response through the management of eicosanoid production (Calder, 2009).

Eicosanoids, such as prostaglandin, thromboxanes, and leukotrienes, are products of arachidonic acid. These eicosanoids are the prime mediators in regulating the intensity of inflammation in the body (Calder, 2009). Therefore, omega-6 fatty acids produce pro-inflammatory responses. Conversely, omega-3 fatty acids have anti-inflammatory properties. They decrease the production of eicosanoids, increase anti-inflammatory resolvins through EPA and DHA, and decrease the production of pro-inflammatory cytokines, such as IL-1β and TNF-α (Calder, 2010). When more omega-3 fatty acids are incorporated into the diet, greater amounts of EPA and DHA are incorporated into the phospholipid layer of the cell (Chapkins et al., 1991), and when more EPA and DHA are present, less arachidonic acid is present in the cell, suppressing eicosanoid synthesis. Therefore, lowering the omega-6:3 fatty acid ratio has been shown to lessen the intensity of the inflammatory response allowing the body to allocate energy and nutrients away from stimulating the immune system and more towards growth performance, especially during a health challenge (Liu et al., 2003; Duan et al., 2014).

The benefits of incorporating omega-3 fatty acids on growth performance can be variable. It is thought that the benefit omega-3 fatty acids bring is only present during a disease challenge. Li et al. (2014) observed no differences in growth performance with the incorporation of omega-3 fatty acids, sourced from a marine omega-3 product, when there was no bacterial or environmental challenge present. These results are like those of experiment 1, where no differences in overall growth performance were observed with increasing levels of omega-3 fatty acids in the diet. Liu et al. (2003) observed no differences in growth performance with the incorporation of omega-3 fatty acids, sourced via fish oil, prior to an LPS challenge. However, pigs fed diets containing fish oil had improved ADG and ADFI after pigs were administered an LPS challenge. These results reflect those of experiment 2, where pigs fed increasing levels of omega-3 fatty acids had increased ADG, ADFI, and G:F once the prevalence of viral shedding of PRRSV increased, as evidenced by the PRRSV-positive oral fluid results on day 21. After PRRSV was detected, a linear benefit in growth performance was observed with increasing levels of omega-3 fatty acids. The improvement found after the viral challenge resulted in increased overall growth performance and reduced total removals and mortality. Huber et al. (2018) explained that when the omega-6:3 fatty acid ratio is reduced, through the addition of omega-3 fatty acids, energy, and other nutrients can be allocated more towards growth performance and less towards maintenance because less energy is needed to mediate inflammation. We speculate that the benefits observed in experiment 2 are thought to be because pigs were fed omega-3 fatty acids long enough before viral challenge which possibly dampened the inflammatory response resulting from PRRSV. However, in experiment 3, there was no improvement in overall growth performance in pigs fed diets containing an increase in omega-3 fatty acids. Because a large portion of pigs in this experiment were positive for PRRSV at arrival at the nursery, there might not have been enough time for omega-3 fatty acids to enrich the cell and influence immune response.

The activation of the immune system during a health challenge includes many interactions between inflammatory responses, different cell types, and antigens. The complexity of the immune system makes it almost impossible to study all at once. Therefore, researchers analyze different components separately (Teitelbaum and Walker, 2001). Lipopolysaccharide is a common and practical model to use to evaluate the overall acute phase immune response (Llamas Moya et al., 2006). Lipopolysaccharide is an endotoxin found in the outer membrane of Gram-negative bacteria. During an LPS challenge, macrophages, and monocytes produce pro-inflammatory cytokines, which can lead to an induced fever and a reduction in feed intake (Carroll et al, 2003).

IL-1β is a pro-inflammatory cytokine responsible for several mechanisms as part of the immune response. Elevated IL-1β concentrations lead to the activation of the hypothalamic-pituitary-adrenal axis, which produces prostaglandin E2, resulting in fever (Song and Horrobin, 2004). Liu et al. (2003), observed a decrease in plasma IL-1β concentrations in pigs fed diets containing omega-3 fatty acids, sourced by fish oil, compared to pigs fed diets containing corn oil. The increase in dietary omega-3 fatty acids is thought to have led to a decrease in eicosanoid production and, therefore, less production of pro-inflammatory cytokines. In experiment 1 of our studies, there were no differences observed in IL-1β concentrations in pigs fed diets containing increasing levels of omega-3 fatty acids in the diet.

TNF-α is another pro-inflammatory cytokine that induces fever during a health challenge by activating the hypothalamic-pituitary-adrenal axis (Wright et al., 2000). Carroll et al. (2003) observed a decrease in TNF-α concentrations in the serum with increasing levels of omega-3 fatty acids, sourced via menhaden fish oil, compared to pigs fed diets containing corn oil. Similarly, Gaines et al. (2003) observed a reduction in TNF-α serum concentrations with the addition of omega-3 fatty acids, sourced by menhaden fish oil, in the diet compared to pigs fed diets containing corn oil. However, the results from experiment 1 did not find a similar response, as increasing omega-3 fatty acids in the diet did not influence TNF-α in the serum compared to the baseline concentration.

The stimulation of a febrile response, due to an LPS challenge, can occur as soon as 15 min post LPS challenge (Wyns et al., 2015). When an LPS challenge is administered, the macrophages produce pro-inflammatory cytokines that stimulate a fever (Johnson and von Borell, 1994). Huber et al. (2018) observed a reduced body temperature 2 h post-LPS challenge compared to controls in pigs fed diets containing omega-3 fatty acids, via fish oil. However, in experiment 1, increasing dietary omega-3 fatty acids had no effect on body temperature post-LPS challenge. Body temperatures did increase 2 h post-LPS challenge and then gradually began to decrease back to normal levels until pigs were back to baseline body temperatures ~12 h post-LPS challenge. These results indicate that the LPS challenge was executed properly; however, no differences in body temperature or cytokine production in the serum were observed.

Though there are many studies confirming the benefits of omega-3 fatty acids on immune function during a health challenge, there were no benefits observed during the LPS challenge in experiment 1. One hypothesis that could explain this is that the source of omega-3 fatty acids used for all the current experiments was less efficient at being converted to EPA and DHA. Menhaden fish oil, an alternative source of omega-3 fatty acids used in other trials, is highly concentrated with EPA (Ratnayake et al., 1988). Past research observed a reduction in pro-inflammatory cytokines and body temperature with increasing levels of dietary omega-3 fatty acids during an LPS challenge when the source of omega-3 fatty acid was fish oil-based (Carroll et al., 2003; Gaines et al., 2003; Liu et al., 2003; Huber et al., 2018). Though the Nannochloropsis algae in O3 trial feed contains high levels of EPA, it may not have provided enough EPA and DHA to impact inflammation and the febrile response during the short-lived LPS challenge in experiment 1. Therefore, increasing omega-3 fatty acids in the diet did not impact pro-inflammatory cytokines and body temperature during the LPS.

PRRSV is one of the most impactful pathogens affecting swine production globally (Montaner-Tarbes et al., 2019). The viral envelope glycoproteins are the first to encounter host cell receptors to initiate infection and stimulate the immune system (Shi et al., 2015). Once infected, typical symptoms of PRRSV virus include severe respiratory disease in newborns and growing pigs and reproductive failure in sows. However, different PRRSV strains and immune status of host cells can play a role in the severity of the infection (Lunney et al., 2016). The innate immune system is the first response to prevent viral replication of PRRSV. The goal is to stimulate a strong adaptive immune response to fight against the infectious agents PRRSV virus enriches within the host cell (Montaner-Tarbes et al., 2019). Additionally, PRRSV virus has mechanisms that suppress the production of cytokines that help strengthen the innate immune response (Van Reeth et al., 1999).

Polyunsaturated fatty acids have been shown to alter responses depending on the type of disease evaluated and the omega-6:3 ratio of the diet. Studies by Walter (2019) concluded that the competency of PUFA, either omega-6, omega-3, or the ratio of the two, depends on the dosage, the amount of time given, and pathogen present. However, little research has been done on the effects of supplementing PUFAs on respiratory health in swine. As previously stated, results from experiment 2 indicated that once pigs tested positive for PRRSV virus on day 21, an improvement in growth performance was observed in pigs fed increasing levels of dietary omega-3 fatty acids. This ultimately led to an improvement in overall growth performance and a reduction in total removals and mortality. However, in experiment 3, no differences in growth performance were observed with the inclusion of omega-3 fatty acids in the diet of pigs that tested positive for PRRSV virus for the duration of the nursery period. This supports a component of the conclusion stated by Walters et al. (2019), that omega-3 fatty acids might need to be fed for a certain period of time before eliciting a benefit. This would allow more EPA and DHA to enter the phospholipid layer of cells, which would improve the immune system and develop cells that are better prepared for a health challenge, like PRRSV. However, further research is needed to determine the amount of time needed to enrich cells with EPA and DHA to improve immune responses and prepare the host cells for invading diseases and infections. Potential research could be conducted to supplement omega-3 fatty acids into gestation diets of PRRSV-positive sow flows, to pass on the benefits of EPA and DHA to subsequent offspring and better prepare host cells and the immune system before pigs are exposed to circulating infections and diseases.

Diagnostic testing using real-time PCR is a common method to detect PRRSV (Trevisan et al., 2019). When interpreting the results of a PCR assay, a higher cycle threshold (Ct) value means that there is less detectable viral genomic material present. In contrast, a lower Ct value means that there is more viral genomic material present in the sample. In experiment 3, there was a highly significant, positive correlation between ADG and Ct values for oral fluids collected 1-wk post-placement into the nursery and, also a significant, positive correlation between ADFI and Ct values for oral fluids collected 1-wk post-placement. These results suggest that as Ct values for oral fluid samples collected early in the nursery turn decrease (more viral RNA present), so do ADG and ADFI. There was a highly significant, negative correlation between total removals and mortality and Ct values for oral fluids collected 1-wk post-placement and a significant negative correlation between total removals and mortality and Ct values for oral fluids collected 3-wk post-placement. These results indicate that as Ct values decrease (greater viral RNA present), total removals and mortality increase. There was no evidence of a correlation between growth and removals and mortality for oral fluid samples collected later in the nursery stage. This could be partially explained by the fact that all oral fluid samples collected at 3- and 4-wk post-placement were PRRSV-positive with Ct values ranging from 27 to 36, whereas oral fluid samples collected 1- and 2-wk post-placement included PCR-negative samples which assumed a Ct value of 40, which was the PCR upper detection limit. These results illustrate that high levels of PRRSV genetic material in oral fluid samples early in the nursery phase result in reduced gain and feed intake as well as greater removals and mortality. It is also important to consider that all pigs used in experiment 3 were sourced from PRRSV-positive sow farms. Magalhaes et al. (2022) explains that there is a strong association between the health of the sow farm and downstream pig performance and mortality and the presence of PRRSV in the sow farm can be a major risk factor on wean-to-finish mortality. The correlation between PRRSV CT and growth performance we observed supports this research. Further research is needed to better explain the relationship between Ct values, growth performance, and mortality rate to understand whether interventions could influence viral shedding and how that could make an impact on growth performance during a health challenge.

## Conclusion

In summary, increasing omega-3 fatty acids in the diet, through the inclusion of O3 trial feed, did not improve growth performance or immune response in healthy pigs given an LPS challenge. This is thought to be due to the high health status of pigs used and O3 trial feed not providing enough EPA and DHA to influence the immune response during a short LPS challenge. If omega-3 fatty acids are fed before a natural PRRSV break, growth performance, immune response, and mortality can be improved during the health challenge. It appears that the inclusion of omega-3 fatty acids in the diet will not be beneficial to growth, immune response, or mortality if fed after a health challenge outbreak. Research on the timing of omega-3 fatty acid supplementation and onset of a disease challenge needs to be evaluated.
